# Immune-profiling of SARS-CoV-2 viremic patients reveals dysregulated innate immune responses

**DOI:** 10.3389/fimmu.2022.984553

**Published:** 2022-11-09

**Authors:** Xiaoming Sun, Ce Gao, Ke Zhao, Yanhui Yang, Yelizaveta Rassadkina, Jesse Fajnzylber, James Regan, Jonathan Z. Li, Mathias Lichterfeld, Xu G. Yu

**Affiliations:** ^1^ Department of Immunology and Pathogen Biology, School of Basic Medical Sciences, Hangzhou Normal University, Hangzhou, China; ^2^ Ragon Institute of MGH, MIT and Harvard, Boston, MA, United States; ^3^ Infectious Disease Division, Massachusetts General Hospital, Boston, MA, United States; ^4^ Infectious Disease Division, Brigham and Women’s Hospital, Boston, MA, United States

**Keywords:** SARS-CoV-2, COVID-19, viremia, RNAseq, interferon stimulated genes, sensing

## Abstract

SARS-CoV-2 plasma viremia has been associated with severe disease and death in COVID-19. However, the effects of viremia on immune responses in blood cells remain unclear. The current study comprehensively examined transcriptional signatures of PBMCs involving T cells, B cells, NK cells, monocytes, myeloid dendritic cells (mDCs), and plasmacytoid dendritic cells (pDCs) respectively, from three different groups including individuals with moderate (nM), or severe disease with (vS) or without (nS) detectable plasma viral load. Whole transcriptome analysis demonstrated that all seven immune cell subsets were associated with disease severity regardless of cell type. Supervised clustering analysis demonstrated that mDCs and pDCs gene signatures could distinguish disease severity. Notably, transcriptional signatures of the vS group were enriched in pathways related to DNA repair, E2F targets, and G2M checkpoints; in contrast, transcriptional signatures of the nM group were enriched in interferon responses. Moreover, we observed an impaired induction of interferon responses accompanied by imbalanced cell-intrinsic immune sensing and an excessive inflammatory response in patients with severe disease (nS and vS). In sum, our study provides detailed insights into the systemic immune response to SARS-CoV-2 infection and reveals profound alterations in seven major immune cells in COVID-19 patients.

## Introduction

Over six million deaths globally are caused by SARS-CoV-2 infection (1). Recent studies indicated that approximately 5 to 10% progress to critical disease and develop acute respiratory distress syndrome (ARDS), and higher fatality rates have been observed in elderly individuals with comorbidities infected with the Wuhan strain (2–4). Recent studies have demonstrated that SARS-CoV-2 plasma viremia in hospitalized patients is associated with severe disease and death (5–7). Although substantial progress has been made toward understanding SARS-CoV-2 immune responses and pathogenesis in different disease severity settings using single cell or bulk RNA sequencing approaches (8, 9), very little is known about immune responses in COVID-19 patients who have detectable plasma viral load.

In general, the viremic phase of viral infections is frequently correlated with the severity of the disease and worse clinical outcomes, such as rhinoviruses (10), respiratory syncytial virus (RSV) (11), adenovirus (12), SARS-CoV (13), and MERS (14). Previous studies found that up to 79% of serum samples have detectable SARS-CoV RNA during the first week of infection, and the rates were similar in MERS (15). We detected 27% of patients with viremia in our cohorts, and other studies have shown detectable plasma SARS-CoV-2 RNA ranging from 0 to 73%, which consistently revealed that it was associated with worse clinical outcomes (15–17). However, the mechanisms behind this association remain elusive. Recently, one study highlighted a cascade of vascular and tissue damage associated with SARS-CoV-2 plasma viremia (6).

Immune dysfunction was observed in patients with COVID-19. Plasma proinflammatory cytokines, including IL-6 and tumor necrosis factor (TNF), and several chemokines, have been detected at elevated concentration in patients with COVID-19 and were associated with disease severity (18). In general, Type I interferons (IFNs) are crucial for restricting viral replication through type I IFN receptor signaling. However, it has been shown that severe COVID-19 patients expressed minimal amounts of IFNs in the peripheral blood, which is in contrast to what has been observed in patients infected with highly pathogenic influenza viruses (19, 20). In addition, interferon dysfunction was also associated with innate defects in TLR-3 and IRF7-dependent type I IFN production (21) and with preexisting neutralizing autoantibodies to type I IFN (22). Innate immune cells act as the first line of defense to activate signaling pathways resulting in the expression of anti-viral molecules, including interferons (IFNs), interferon-stimulated genes (ISGs), and inflammatory chemokines and cytokines (23). Studies have been shown that infected myeloid dendritic cells failed to trigger a significant production of anti-viral cytokines such as IFN-α, IFN-β, IFN-γ upon SARS-CoV-2 infection, while promoting a moderate increase of pro-inflammatory cytokines including TNF-α and IL-6 (24). In addition, recent studies also showed that infected circulating monocytes can induce acute inflammatory responses and cause cytokine storm, tissue-damage, and cell death (25). Furthermore, a recent study has shown that pDCs are resistant to SARS-CoV-2 infection but can be efficiently activated by the virus (26, 27).

In the present work, we have conducted a parallel, unbiased transcriptional profiling analysis of CD4 T cells, CD8 T cells, B cells, natural killer (NK) cells, monocytes, myeloid DCs (mDCs) and plasmacytoid DCs (pDCs) from three different disease severity groups with healthy controls. Our data show that COVID-19 disease severity is associated with the induction of interferon-stimulated genes (ISGs). In addition, antigen-presenting cells showed imbalanced type I interferon sensing pathways and proinflammatory responses. Together, these data highlights important roles of type I interferon in immune defense during acute SARS-CoV-2 infection in humans.

## Material and methods

### Participant enrollment and sample collection

We enrolled COVID-19 patients at Massachusetts General Hospital. The blood sample was collected and processed within 24 h of collection by the Ragon Institute. PBMC samples were used under protocols approved by the Mass General Brigham Institutional Review Board. Clinical information and demographical characteristics of study patients were collected from the clinical medical record. The disease severity was defined by oxygenation status and hospitalization status to classify the disease severity as mild, moderate, severe and critical by clinicians (**Table S1**). Study patients gave written informed consent to participate in accordance with the Declaration of Helsinki.

### Markers of inflammation and disease severity

Levels of CRP, D-dimer, Trop, bFGF, and absolute lymphocyte count were recorded from the electronic medical record from Massachusetts General Hospital. Thirty-five additional markers of inflammation were evaluated in plasma by the Luminex xMAP assay (ThermoFisher): epidermal growth factor, Eotaxin, fibroblast growth factor-basic, granulocyte colony-stimulating factor (CSF), granulocyte-macrophage CSF, hepatocyte growth factor, IFN-α, IFN-γ, IL-1α, IL-1β, IL-1RA, IL-2, IL-2R, IL-3, IL-4, IL-5, IL-6, IL-7, IL-8, IL-9, IL-10, IL-12 (p40/p70) IL-13, IL-15, IL-17A, IL-17F, IL-22, IP-10, MCP-1, MIG, MIP-1α, MIP-1β, RANTES, tumor necrosis factor-α, and vascular endothelial growth factor as described previously (5).

### PBMC processing

The EDTA anticoagulant blood was centrifuged at 2600rpm for 15min at room temperature to collect plasma and store at -80, the remaining blood was diluted with HBSS at 1:1ratio and layered to 14ml Ficoll-plus(sigma), centrifuge at 1500 rpm for 30min with brake on. The PBMC layer was collected and washed with PBS twice, cells were counted and stored in liquid nitrogen for a long-term storage.

### SARS-CoV-2 viral load quantification

Levels of SARS-CoV-2 viral load were quantified using the US CDC 2019-nCoV_N1 primers and probe set as previously described for wild-type virus (5). Briefly, the supernatant was removed and TRIZOL Reagent (Thermo Fisher, USA) was added to the pellets and then incubated on ice, followed by chloroform (MilliporeSigma, USA). The mixtures were separated by centrifugation at 21,000 x g for 15 minutes at 4°C, and subsequently the aqueous layer was removed and treated with an equal volume of isopropanol (MilliporeSigma, USA). GlycoBlue Coprecipitant (ThermoFisher, USA) and 100 µL 3M sodium acetate (ThermoFisher, USA) were added to each sample. RNA was pelleted by centrifugation at 21,000 x g for 45 minutes at 4°C. The supernatant was discarded and the RNA was washed with cold 70% ethanol and resuspended in DEPC-treated water. 1× TaqPath™ 1-Step RT-qPCR Master Mix kit (ThermoFisher) was used to quantify viral load, the CDC N1 forward and reverse primers, and probe, and an in-house N1 standard curve was used to calculate viral copy number. SARS-CoV-2 viral loads below 40 RNA copies/ml were categorized as undetectable and set at 1.0 log10 RNA copies/ml.

### Flow cytometry and cell sorting

For cell sorting, PBMCs were stained with antibodies from Biolegend against CD3 (clone OKT3), CD4 (clone OKT4), CD8 (clone SK1), CD19 (clone HIB19), CD56 (clone HCD56), CD16 (clone B73.1), CD14 (clone HCD14), HLA-DR (clone L243), CD11c (clone 3.9), CD123 (clone 6H6), and Live/Dead fixable violet stain (BVD, Life Technologies). Cell sorting for total CD4 T cells (CD3^+^ CD4^+)^, total CD8 T cells (CD3^+^ CD8^+^), total monocytes (CD3^−^ CD19^−^CD14^+^), total NK cells (CD3^−^ CD19^−^ CD14^−^CD56^bright/+/dim^CD16^-/+^), total B cells (CD3^−^ CD14^−^ CD19^+^), total pDCs (CD3^−^CD14^−^CD19^−^CD56^−^HLA-DR^+^ CD11c^−^ CD123^+^), and total mDCs (CD3^−^CD14^−^ CD19^−^CD56^−^ HLA-DR^+^ CD11c^+^ CD123^−^),were performed using BD FACSAria Fusion by the Ragon Institute Imaging Core Facility and resulted in the isolation of these seven subsets with the defined phenotypic characteristics at >95% purity(**Figure S1**). For entry receptor staining, ACE2(clone 535919), CD26(clone BA5B), CD13(clone WM15), CD147(clone HIM6), CD249(clone 2D3) and TMPRSS2(clone 1038105).

### Transcriptional profiling by RNA sequencing

The sorted seven cell populations were used for RNA extraction using a commercial PicoPure RNA Isolation Kit (Thermo Fisher Scientific) and DNA was removed by in-column DNAse treatment(RNase-Free DNase Set, Qiagen). The RNA purity and concentration was measured by Nanodrop 2000. RNA-Seq libraries were generated using the SMART-seq2 protocol as described in a previous study (28). Briefly, a total of 10 ng of RNA and 1 μl 1:10^6^ dilution of external ERCC RNA spike-in control as input material for all cell subsets. Whole transcriptome amplification and tagmentation-based library preparation was performed using the SMART-seq2 protocol, followed by sequencing with 35bp Pair-end using a 75-cycle kit on a NextSeq 500 instrument (Illumina, CA). The raw reads were aligned to the Hg38 human genome database and SARS-CoV-2 Wuhan-Hu-1 strain was used as reference for virus alignment.

### Computational data analysis of RNA-Seq data

DESeq2 implemented in the Bioconductor/R-project package to detect DEGs was used to calculate FDR-adjusted p-values (29). IPA was used to functionally categorize DEGs, including canonical pathways, upstream regulators and disease&functions. IPA is a software program which can analyze the gene expression patterns using a build-in scientific literature-based database (QIAGEN Inc.). Pathway analysis was carried out using gene set enrichment analysis (GSEA) (30), and Networkanalyst Reactome(https://www.networkanalyst.ca/) (31). Principal component analysis (PCA) and Linear discriminant analysis (LDA)plots were generated using the package in R.

### Interferon stimulated genes and ISG score

Human Interferon stimulated genes were downloaded from the Interferome v2.0 database. An ISG score was calculated based on the mean expression of six ISGs (IFI44L, IFI27, RSAD2, SIGLEC1, IFIT1, and ISG15) as described previously (19).

### Statistics

Statistical significance between the different subsets was tested using Mann–Whitney U-tests or Wilcoxon matched-pairs signed-rank tests. Experiments were conducted once with each described sample unless indicated otherwise in the text. NS, not significant; *:p < 0.05, **:p < 0.01, ***:p < 0.001, ****:p < 0.0001. Statistical significance was analyzed in GraphPad Prism v7, and RNA-seq data analysis was performed in R v3.5.2.

## Results

### Characteristics of subgroups of COVID-19 patients with different severity

**Figure 1:**
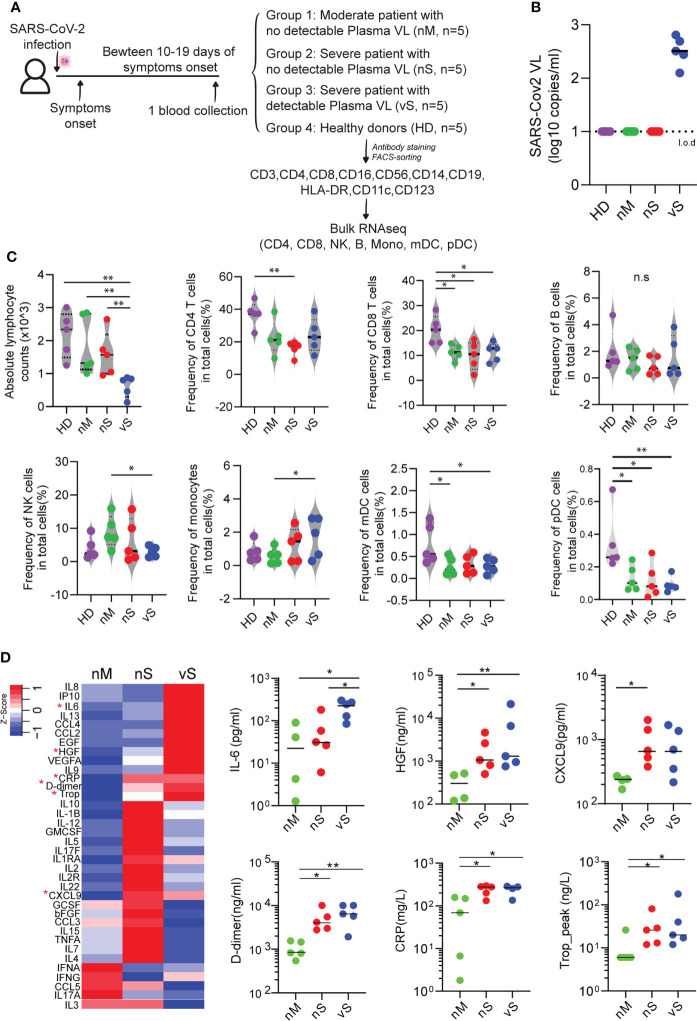
Cell frequency and cytokines changes observed in participants with different disease severity. **(A)** Overview of experimental design. PBMCs from three groups of COVID-19 patients with moderate (nM, n=5), a severe disease without detectable plasma viral load (nS, n=5), severe disease with detectable plasma viral load (vS, n=5), and healthy donors (HD, n=5) were collected. Seven major cell types were sorted and subjected to bulk RNAseq. **(B)** Levels of SARS-CoV-2 plasma viral loads were measured by qRT-PCR and the dashed line indicated the limit of detection (l.o.d). **(C)** Violin plots of 7 major cell lineage composition in PBMCs from the nM, nS, vS, and HD groups by flow cytometry analysis. **(D)** Heat map showed secretion level of 36 cytokines in plasma from different study groups, and any group that shows statistical significance were highlighted in red star and also shown in the dot plot. A p-value was calculated by the Kruskal-Wallis test. *: p < 0.05, **: p < 0.01, n.s., not significant.

Current studies suggest that SARS-CoV-2 plasma viremia can be associated with a higher risk for severe disease and death in COVID-19 cases, but detailed mechanisms are not well defined. To gain systemic insights into the immune response caused by SARS-CoV-2 infection in different disease severity conditions, we conducted transcriptional profiling experiments to characterize gene expression changes in seven immune cell populations (CD4 T cells, CD8 T cells, B cells, NK cells, monocytes, mDCs, and pDCs) from the peripheral blood of three different groups, including individuals with moderate disease (nM, n=5), with severe disease without detectable plasma viral load (nS, n=5), and with a severe disease with detectable plasma viral load (vS, n=5), and cells from five gender- and age-matched healthy individuals (HD, n=5) were treated identically and were used as reference samples (**Figures 1A, B**). The clinical characteristics of these four study groups were described in **Supplementary Table 1** and disease severity was classified by oxygenation status and hospitalization status by clinicians at the Brigham and Women’s Hospital and Massachusetts General Hospital. Consistent with previous studies, we observed the vS group had significantly lower absolute leukocyte counts than the other two groups suggesting more severe lymphopenia (**Figure 1C**). To allow for an in-depth analysis of cell subset frequency, we performed phenotyping of surface marker expression by flow cytometry. We observed that the frequency of T cell populations in COVID19 patients were significantly lower compared to healthy controls ; these changes were more pronounced for in CD8^+^ T cells, consistent with previous reports in a larger cohort (32, 33). Similarly, there were significantly lower frequencies of mDCs and pDCs in COVID-19 patients compared to patients with healthy controls. We further found there are no changes in B cell frequency. In contrast, NK cell frequency only significantly lower in the vS group, and monocytes were significantly higher in the vS group compared to other groups (**Figure 1C**). In addition, a previous study from our study cohort has shown that higher plasma viral loads were significantly associated with inflammation (5). In line with the previous observation (5), we also observed that the plasma levels of inflammatory markers such as IL-6, D-dimer, HGF, and CRP were correlated with disease severity (**Figure 1D**).

### Systemic inflammatory responses in COVID-19

**Figure 2:**
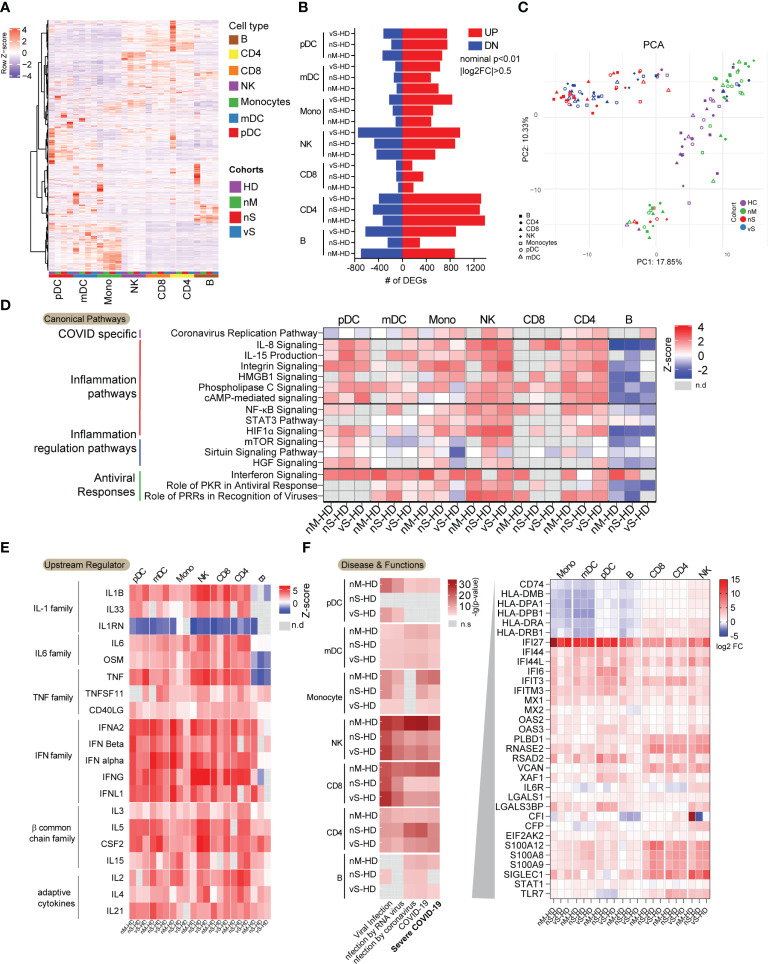
Dysregulated transcriptional immune profiles in COVID-19 infection. **(A)** Heatmaps demonstrated transcriptional patterns of highly variable genes (n=3452) among the three groups of COVID-19 patients and the non-infected individuals in indicated immune cell subsets. The colored square on the bottom of the heat map indicated the cell types and study cohort information. **(B)** Bar diagram indicating the total number of upregulated (red) and downregulated (blue) genes in indicated cell populations from three groups of COVID-19 patients relative to non-infected individuals (|FC| = 0.5, nominal p< 0.01). Each pathway was grouped according to its functions. **(C)** PCA analysis of global transcriptional signatures from indicated cell populations derived from COVID-19 group, nM(green), nS(red), vS(blue), and healthy control (HD, purple). **(D)** Common Canonical pathways inferred by IPA from DEGs between COVID-19 patients and non-infected individuals in indicated immune cell subsets. Pathways were grouped by functions. Red and blue denote functional pathways predicted to be up or downregulated, respectively; gray indicates indeterminate directional. **(E)** Common predicted upstream regulators for genes differentially expressed between indicated immune cell subsets from COVID-19 patients and non-infected individuals. Color coding reflects a z-score, indicating activation or inhibition of the upstream regulators. Missing values were in gray. **(F)** Diseases and Functions inferred by IPA involving virus infections were shown. The red gradient indicated the p-value and missing value in gray. The genes related to severe COVID-19 were listed, and expression levels (log2FC) were shown in indicated immune cell types.

To better understand how the cellular response to SARS-CoV-2 infection might shape the disease course, we next analyzed transcriptomic data for seven immune cell types in detail. We observed that gene expression signatures differed profoundly on a global transcriptional level among the individual cell populations (**Figures 2A**, **S1A**). Specifically, CD8 T cells showed relatively minor transcriptional differences between COVID-19 patients and controls, with less than 400 transcripts meeting our criteria for differential expression. However, in the remaining cell types, SARS-CoV-2 infection was associated with a markedly altered transcriptional profile, with numbers of differentially expressed genes (DEGs) ranging from 700 in mDCs to more than 1800 in CD4 T cells (**Figure 2B**). Notably, the vS group in all cell types tended to have more DEGs when compared with healthy controls than other COVID-19 groups. Next, we performed principal component analysis (PCA) to compare the effects of infection among different cohorts. Clearly, we observed that the nM patients grouped together with the HD patients, while being very different from the two severe groups, which also grouped together. Unexpectedly, all types of cells among PBMCs clustered together according to the disease groups instead of cell types, suggesting that seven major blood immune cells may be influenced by common inflammatory mediators regardless of cell types (**Figure 2C**). Indeed, computational canonical pathway analysis of DEGs predicted that key common functional entities involved in inflammation pathways were upregulated except in B cells, these pathways included, IL-8 signaling, IL-15 production, HMGB1 signaling, HIFA signaling. We observed that 7 immune cells mounted antiviral responses, especially within the nM group, in which a slightly higher z-score for interferon signaling was noted compared to the nS and vS groups (**Figure 2D**). Moreover, this computational analysis also predicted that upstream regulators in all cell types involved the inflammatory cytokines such as members of the IL-1 family, the IL-6 family, and the TNF family cytokines, these inflammatory markers were potentially more prominent in the nS and vS groups while the IFN family represented a more dominant predicted upstream regulator in the nM groups suggesting that an imbalance between inflammatory responses and type I interferon responses in patients with severe diseases (**Figure 2E**). We next focused on the genes involved in severe COVID-19, specifically on HLA class II-encoding genes ,which were downregulated in all antigen-presenting cells, including mDC, pDC, monocyte, and B cells from the three groups of COVID-19 patients relative to healthy controls (**Figure 2F**), concordant with other studies (34, 35). Given the profound degree of transcriptional changes and the polyadenylation of the SARS-CoV-2 genome and subgenomic RNA (sgRNA), we sought to determine which cell types were positive for viral RNA. We recovered viral reads from the sequencing data and mapped them to the reference viral genome of SARS-CoV-2 isolate Wuhan-Hu-1 (36). We found there were no reads that mapped to the SARS-CoV-2 genome from any cell subsets, and these results were further confirmed by RT-PCR from total PBMC samples, suggesting that immune cells circulating in blood are not susceptible to SARS-CoV-2 infection *in vivo* despite the detection of low-level expression of the entry receptor ACE2 in monocytes and mDCs (**Figure S1**).

### Type I interferon-driven inflammatory signatures in mDCs and pDCs are characteristics of COVID-19 severity

**Figure 3:**
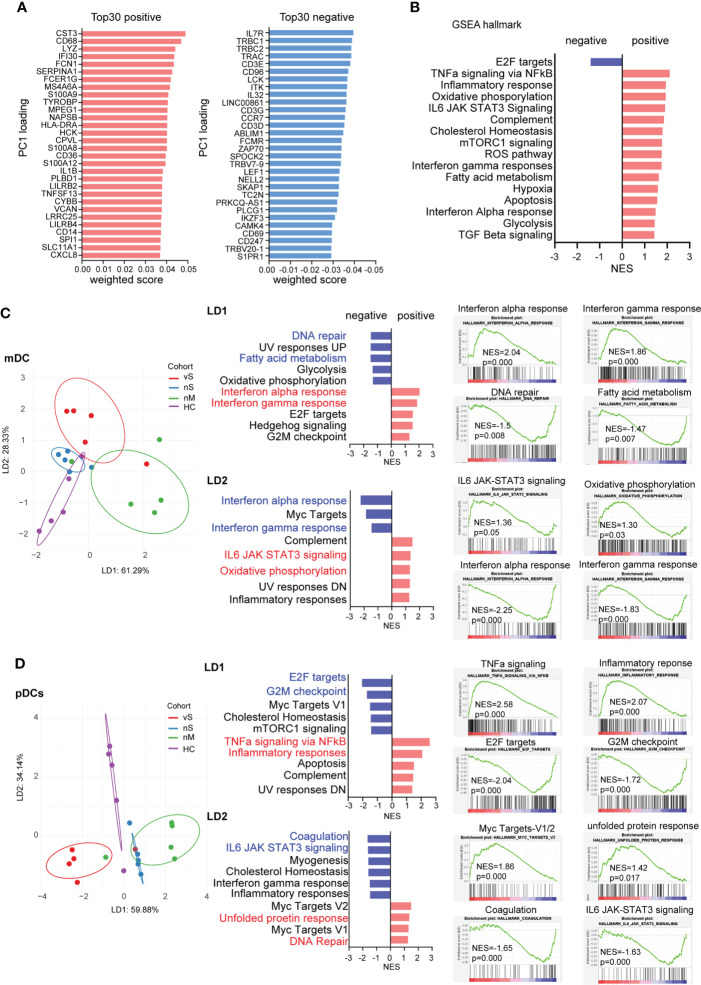
mDCs and pDCs can distinguish COVID-19 patients with different disease severity **(A)**. Top 30 weighted scores for positive (red) and negative (blue) PC1 loading. **(B)** gene set enrichment analysis (GSEA) by using hallmark gene sets, normalized enrichment scores are plotted (FDR p<0.05), and Red bars show the enrichment signatures for a positive weighted score, and bars reflect negative score. **(C)** Linear discriminant analysis on mDCs was used to separate HD (purple), nM (green), nS (blue), and vS (red) groups. Hallmark gene sets were used for GSEA, and the five signatures with strongest enrichment (red) or de-enrichment (blue) are shown. Enrichment plots for the top 2 signatures are shown individually. **(D)** Linear discriminant analysis on pDCs was used to separate HD (purple), nM (green), nS (blue), and vS (red) groups. Hallmark gene sets were used for GSEA, and the five signatures with strongest enrichment (red) or de-enrichment (blue) are shown. Enrichment plots for the top 2 signatures are shown individually.

We next assessed overall differences among COVID-19 patients with different disease severities. The first principal component (PC1) of PCA clearly explained the differences between COVID-19 patients with severe diseases (red and blue dots) and those with moderate disease (green dots), healthy donors (purple dots) (**Figure 2C**). The top 30 positive and negative loading scores from Principal Component 1 (PC1) were plotted, and interestingly, we observed that the most differences were associated with innate immune responses and inflammation-related genes in positive score genes, such as CD68, CD36, CD14, S100A8, S100A9, while the gene list with negative scores related to T cell hyperactivation, including genes encoding for IL7R, IL32, CCR7, ZAP70, CD69, consistent with previous studies showing that T cell hyperactivation is associated with disease severity (**Figure 3A**). A GSEA analysis showed that inflammatory responses, interferon-gamma responses, hypoxia, and apoptosis gene sets were positively enriched in the nM group. In contrast, cell proliferation and cell cycle-related E2F targets gene sets were enriched within the severe patient groups (**Figure 3B**).

To further achieve maximum separation between the four groups, we performed a supervised linear discriminant analysis (LDA). We found different types of cells could be distinctly separated by LD1, LD2, and LD3, but not with disease severity (**Figure S2A**). Thus, we applied LDA to each individual cell type and demonstrated mDC and pDC cells have the best separation among the four groups (**Figures 3C, D**, **S2B**). LD1 in mDCs separated the nM group from the rest of the groups by interferon-alpha responses and interferon-gamma responses that were enriched by GSEA analysis. LD2 distinguished both the nS and vS groups based on IL6 signaling, oxidative phosphorylation, and inflammatory responses from the nM and HD groups. Similarly, LD1 in pDCs showed that the vS group is more enriched with cell cycle-related signature such as G2M checkpoint and E2F targets; in contrast, in LD2, transciptional signatures of pDCs from COVID-19 patients were enriched with inflammatory response, IL6 signaling and, interferon-gamma responses, consistent with other studies (26, 37) (**Figures 3C, D**). In addition, to further illustrate the detailed differences among the nM, nS, and vS groups when compared with HD groups, we visualized the DEGs as heatmaps and also performed a canonical computational pathway analysis by IPA. Overall, the nM had a higher z-score for Interferon signaling when compared with the nS and vS groups, and pathways such as glycolysis metabolism were more enriched in the nS and vS groups compared with the nM group implying that interferon signaling may be associated with better disease outcome; this is consistent with our GSEA results (**Figures S2C-I**). Furthermore, in order to investigate the detailed difference between the nS and vS group, we next calculated the DEGs for mDCs, monocytes and pDCs. IPA pathway analysis suggested that the vS group were enriched in cell cycle-related and cell death pathways such as cell cycle control of chromosomal replication and apoptosis signaling (**Figure S3**).Together, we suggested that three COVID-19 patient groups demonstrated distinct gene signatures and that mDCs, and pDCs were able to separate each COVID-19 patient group distinctly.

### Enhanced interferon response across immune cell populations defines protective immunity in moderate patients

**Figure 4:**
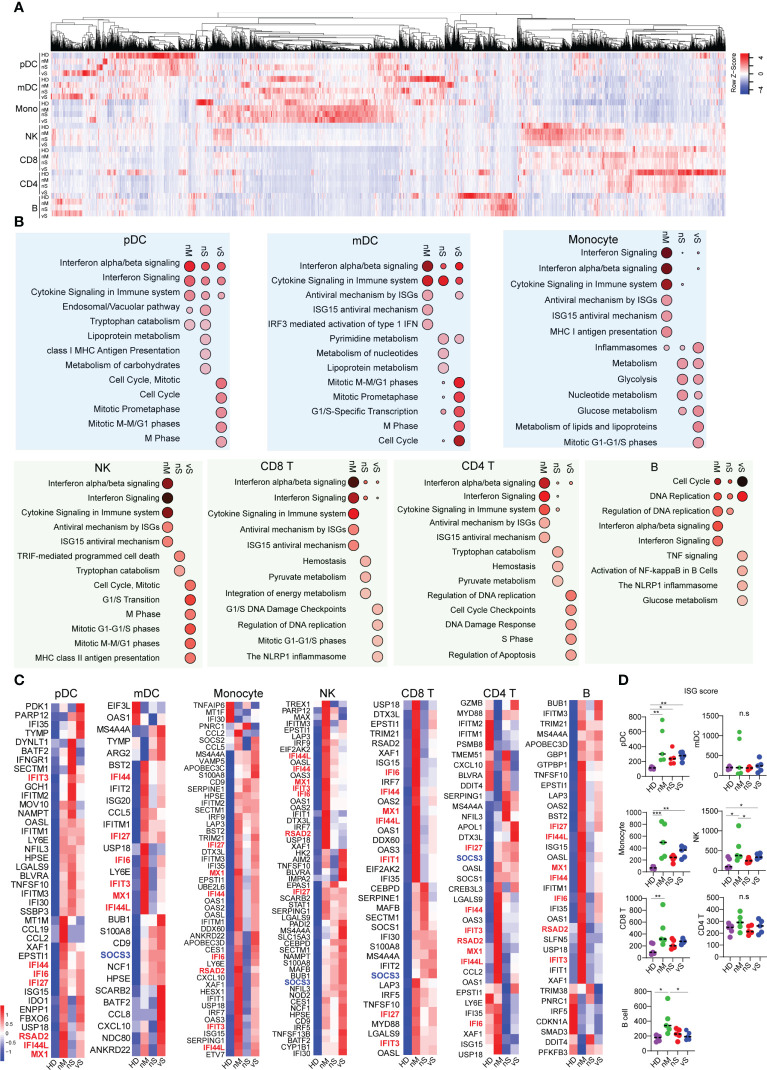
Interferon stimulated genes correlated with disease severity. **(A)** Heatmap reflecting expression intensity of a known gene list of interferon-stimulated genes (ISGs) downloaded from Interferome v2.01 during *in vivo* SARS-CoV-2 infection in different disease cohorts. **(B)** Top 10 enriched Reactome pathways, analyzing by NetworkAnalyst, identified in each cell type when compared with healthy control. The bubble size indicates the gene enrichment ratio of a biological process GO term, with color maps reflecting the FDR value (p. adjust) of the enrichment analysis. **(C)** Heatmap showing the expression of antiviral ISGs that are significantly different in COVID-19 conditions from healthy controls, ordered by hierarchical clustering. Upregulated genes are shown in red, and down-regulated genes are shown in blue. **(D)** ISG score was calculated based on the expression of six genes (IFI44L, IFI27, RSAD2, SIGLEC1, IFIT1, and ISG15) based on TPM transcripts. A p-value was calculated by the Kruskal-Wallis test. *p < 0.05, **p < 0.01, ***p < 0.001; n.s., not significant.

ISGs are the effectors of the initial host antiviral response and are engaged in a wide array of functions in the cell (38). Our DEG analysis suggested that the nM group has better interferon signaling responses than the severe patient group. We next aimed to investigate the Interferon stimulated genes (ISGs) in person with different disease severity. The heatmap showed a similar pattern of gene signatures with DEGs analysis, and detailed individual heatmaps reflected that each cell type revealed dramatic changes compared with the healthy controls (**Figures 4A**, **S4A**). Gene enrichment analysis with the Reactome database revealed a higher enrichment of “interferon-alpha/beta signaling”, “interferon signaling”, “antiviral mechanism by ISGs “ISG15 antiviral mechanism” in all seven immune cell subsets in the nM group. In contrast, we observed that the transcriptional signatures in the nS group were more enriched for metabolism pathways, including “tryptophan catabolism”, “lipoprotein metabolism”,” glycolysis”,” pyruvate metabolism”. Notably, the vS groups were uniquely enriched for cell cycle-related pathways, for instance, “cell cycle, mitotic”, “M phase”, “S phase”, and inflammasome pathways (**Figures 4B**, **S4B**). Specially, we focused on antiviral ISGs expression profiling in each cell type. Assessment of all seven immune cell populations revealed consistent upregulation of many ISGs in the nM groups. For instance, IFI44L, MX1, RSAD2 (Viperin), IFI6, IFI44, IFIT3, IFI27, which are involved in viral containment, showed a higher level in all cell types of the nM group compared with the nS and vS groups. In contrast, we also observed that SOCS3, a negative regulator of type I interferon signaling, was upregulated in mDC, NK, CD4, and CD8 T cells from the vS group (**Figure 4C**). We further computed an ISG-score for each cell population as previously described. Indeed, the ISG score across all cell populations revealed that the COVID-19 groups displayed a higher score than the HD controls and those differences were more profound in the nM groups compared to HD controls (**Figure 4D**). Together, these data revealed a balanced protective induction of ISGs in peripheral immune cells from patients with moderate disease who were able to successfully contain SARS-CoV-2 infection.

### Unbalanced sensing pathways between proinflammatory cytokines and type I Interferon in mDCs, pDCs, and monocytes

**Figure 5:**
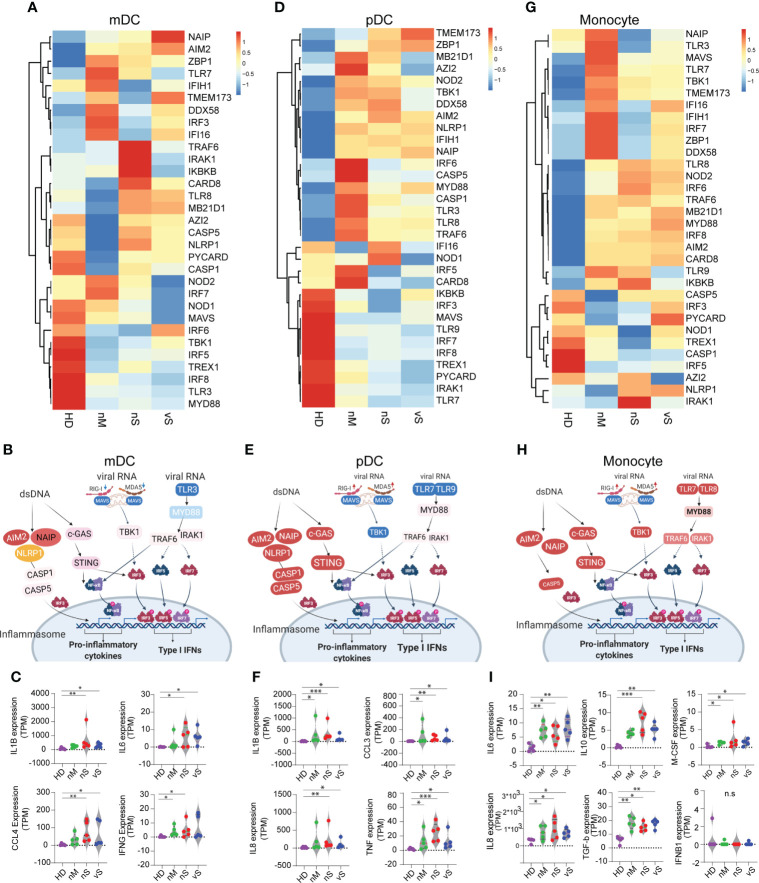
SARS-CoV-2 Infection lead to a shift in immune sensing toward proinflammatory cytokines and impaired type I interferon pathways. **(A, D, G)**. heatmaps showing RNA expression levels of DNA and RNA sensor genes in HD, nM, nS, and vS conditions in mDC **(A)**, pDC **(D)**, and Monocytes **(G)**. **(B, E, H)**. Graphic illustration of key molecule expression changes related to healthy controls in DNA/RNA sensing pathways. Red and blue indicated gene expression to be up- or -downregulated, respectively. Data from mDC **(B)**, pDC **(E)** and Monocytes **(H)** are shown. **(C, F, I)**.Significant differentially expressed downstream effector molecules of proinflammatory cytokines or type I Interferon are listed. mDC (IL1B, IL6, CCL4, and IFNG) **(C)**; pDC (IL1B, CCL3, IL8, and TNF) **(F)**; Monocytes (IL6, IL10, M-CSF, IL8, TGF-b, and IFNB1) **(I)**. A p-value was calculated by the Kruskal-Wallis test. *: p < 0.05, **: p < 0.01, n.s., not significant.

Recent studies discovered that impaired type I interferon response and excessive inflammatory response were associated with disease severity (19, 20). However, the host sensing factors that balance the expression of inflammatory cytokines and type I Interferon are largely unknown. The RNA-sensing arm is activated by RNA viruses, while the DNA-sensing arm is triggered by host damage-associated molecular patterns (DAMPs) released as byproducts of viral reproduction and tissue injury (39). Here we investigated sensor molecules and pathways in antigen-presenting cells, namely mDCs, pDCs, and monocytes. Importantly, we observed dramatic changes among different groups in all three cell types. In mDCs, we found TLR3 viral RNA sensing pathways were downmodulated in all COVID-19 subgroups. In addition, mitochondrial antiviral-signaling protein (MAVS) and NOD1/NOD2 were more downregulated in the vS group compared with others suggesting impaired type I Interferon in severe COVID-19 groups, while NLRP1, AIM2, NAIP expression, involved in different inflammasome pathways, were higher in the nS and vS groups compared with the nM and HD groups further suggesting an imbalance between inflammatory responses and type I Interferon in the severe patient group (**Figures 5A, B**). Indeed, we observed that the transcripts for IL6, CCL4, IL1B, and IFNg are highly expressed in the nS and vS group, but no IFNa/b transcripts were detected (**Figure 5C**). pDCs specialize in endosomal TLR7/9-mediated recognition of viral nucleic acids, and primarily represent type I Interferon producing cells. We observed that TLR7, together with MAVS, TLR9, IRF7, was downregulated in pDCs in the COVID-19 groups but more so in the groups with severe disease. Similar to the mDC subsets, the NLRP1, AIM2, NAIP expression was also highly activated pDCs from the in COVID-19 groups. Subsequently, the expression of proinflammatory cytokines, such as IL1B, CCL3, IL8, and TNF, were significantly upregulated in the pDC subsets (**Figures 5D-F**). Similar trends for a reduced IFN signature and MHC class II expression but increased proinflammatory pathways were found in the monocyte subsets from patients with severe disease compared to moderate disease (**Figures 5G-I**).

## Discussion

In this study, we report a comprehensive transcriptome analysis on seven major peripheral immune cell types from SARS-CoV-2 infected patients with different disease severity. We suggested that type I Interferon and Interferon stimulated genes (ISGs) play protective roles in SARS-CoV-2 infection and indicate that both severe COVID-19 patient groups with/without detectable plasma viral loads are accompanied by a lower type I interferon response in addition to a higher inflammatory response. Taken together, the data presented here suggest that the response to SARS-CoV-2 are characterized by a profound imbalance between type I Interferon activities and inflammatory responses.

Inflammation is a double-edged sword and can result in the development of severe disease (40). Increasing evidence suggest that systemic inflammatory responses play a crucial role in the progression and severity of COVID-19 (19, 41, 42). In this study, we carried out bulk RNA-seq using sorted immune cells from COVID-19 patients with different severity and healthy donors, and hierarchical clustering analysis showed that all sorted cell types were clustered together according to the disease groups rather than cell types as shown in **Figure 2C**, indicating that there is a disease-specific global impact across analyzed cell types. In addition, IPA predicted upstream regulators show that all immune subsets are enriched in proinflammatory cytokines, such as IL-1, IL6, TNF family cytokines in **Figure 2E**. This findings suggest that peripheral blood immune cells are influenced by common inflammatory mediators regardless of cell type. Indeed, recent study from Lee et al. performed scRNA-seq using PBMCs from patients with COVID-19, Flu, and healthy controls (43). Similar to our study, they found peripheral blood immune cells may be influenced by common inflammatory mediators regardless of cell type. Dysregulation of the inflammatory response resulting in a cytokine storm has been proposed as an important factor in the pathogenesis of severe COVID-19. In line with this, a higher level of IL-6 was observed in the vS patients (**Figure 1D**). Moreover, levels of plasma viremia in serum correlated with extremely high levels of IL-6 (5). Therefore, detection of SARS-CoV-2 in serum could thus be a result of leakage from tissues damaged by the inflammatory response.

Type I interferon response constitutes the central first line of defense against viruses (44). The severity of SARS-CoV-2 infection is fueled by the dysregulation of the host immune response primarily by inhibiting type I interferon (IFN) response in acutely infected cells. Both viral and host factors determine the outcome of IFN signaling. For instance, many proteins have been reported to inhibit multiple steps in IFN-I production and signaling mainly involving the MAVS-RIG-I (45, 46) and the cGAS-STING pathways (47, 48). For instance, nsp1, nsp6, nsp13, ORF3a, M, and ORF7b can block STAT1 phosphorylation (49). In addition, viral proteins nsp6, nsp13, ORF7a, and ORF7b suppress STAT2 phosphorylation (50). Furthermore, Q. Zhang et al. identified patients with severe COVID-19 who have mutations in genes involved in the regulation of type I and III IFN immunity (21). Upon sensing of coronaviruses by various pathogen recognition receptors, TLRs (TLR3, TLR7, TLR8, TLR9) and RLRs (RIG-I, MDA5), stimulate the production of proinflammatory cytokines and type I interferons, respectively. In particular, TLR7 plays a critical role in sensing coronaviruses, including SARS-CoV, MERS-CoV, and is required for IFN-a production by pDCs (51). However, we observed that TLR7 receptors in pDCs were significantly downregulated in COVID-19 patients and more obvious in the vS group. In addition, we observed that TLR3 was downmodulated in mDC, while TLR7/8 receptor was upregulated in monocytes. Recent studies also suggested that SARS-CoV-2-infected immune cells, such as monocytes and macrophages, had detectable NLRP1 and AIM2 inflammasomes that recognize cell membrane damage and cytosolic DNA, respectively (52–54). We indeed observed that inflammasome pathways were all activated in mDC, pDC, and monocytes. However, we only analyzed transcriptional data and further work is needed to determine whether the described changes also occur on a protein level and are relevant for functional mechanisms of disease pathogenesis.

Of note, the reactome enrichment analysis showed that the transcriptionl signatures of the vS patient group were highly enriched in cell cycle-related pathways compared with the other groups (**Figure 4B**), consistent with the GSEA hallmark analysis indicating that E2F targets were enriched in the vS group (**Figure 3C**). These results suggested that the cell cycle was arrested in the vS group. There are several possible interpretations. First of all, ours and the transcriptome analysis of others suggested that SARS-CoV-2 infection induce a systemic inflammatory cytokine responses in patients with severe disease (43) and previous studies confirmed that higher levels of tumor necrosis factor (TNF)α, interleukin (IL)-6, and other pro-inflammatory cytokines could induce lymphocyte deficiency (55, 56). Secondly, inappropriate cell hyperactivation could results in a G2/M cell cycle arrest and cell death (57). In addition, studies have also shown that the viral N protein and p53 of host proteins that are the key factors of coronavirus-mediated cell cycle regulation (58, 59).

There are several limitations to this study. First, the sample size is comparatively small, and we only analyzed 7 major cell types because of the limited cell numbers from each sample, although it was sufficient to find different patterns in the gene expression with different disease severity. Second, the data provided are mainly derived from the blood but do not reflect immune responses within the lung. Third, we only analyzed transcription data but no additional validation data. Also, the longitudinal data evaluating plasma viremia of SARS-CoV-2 on viremia is lacking. Therefore, future studies with longitudinal samples from more patients with COVID-19 and a combination of different validation approaches may help to determine the cause-and-effect relationships between the immune characteristic of different cell types and disease outcomes.

## Data availability statement

The RNA-Seq data in this study has been deposited to the NCBI GEO and are available under accession number GSE216529 and GSE132228.

## Ethics statement

The studies involving human participants were reviewed and approved by Mass General Brigham Institutional Review Board. The patients/participants provided their written informed consent to participate in this study.

## Author contributions

XS and XGY conceived of and designed the study. XS, CG, KZ, YY, YR, JF, JR performed data analysis and conducted experiments. XS, ML, and XGY wrote and edited the manuscript. XGY and JL organized and provided clinical samples. All authors contributed to the article and approved the submitted version.

## Funding

XGY is supported by NIH (Grants AI098484, HL126554, AI116228 and HL134539). XS is supported by grant 4255C50222204027. The funders had no role in the study design, data collection, data analysis, or preparation of the manuscript.

## Acknowledgments

We thank members of the Massachusetts consortium on Pathogen Readiness Specimen Working Group for blood samples.

## Conflict of interest

The authors declare that the research was conducted in the absence of any commercial or financial relationships that could be construed as a potential conflict of interest.

## Publisher’s note

All claims expressed in this article are solely those of the authors and do not necessarily represent those of their affiliated organizations, or those of the publisher, the editors and the reviewers. Any product that may be evaluated in this article, or claim that may be made by its manufacturer, is not guaranteed or endorsed by the publisher.

## Supplementary material

Supplementary Figure 1
**(A)**The flow cytometry gating strategy of seven population and purity. **(B)** the expression level of putative SARS-CoV-2 entry receptors, including ACE2, CD13, CD26, CD147, CD249, and TMRPSS2, were accessed by flow cytometry. The left panel shows the representative histogram plots of antibody staining gating on monocytes. A Fluorescence Minus One (FMO) control showed in blue and the sample in red. The right panel summarizes each receptor expression level in different cell types (n=20).

Supplementary Figure 2 Transcriptional signatures of immune cell subpopulations during naturally acquired COVID-19 diseases. **(A)** Linear discriminant analysis of immune responses for various groups with all seven immune cell types. **(B)** Linear discriminant analysis of immune responses for each cell type for different study groups. **(C-I)** Heatmaps demonstrating transcriptional patterns of differentially expressed genes (DEGs) between COVID-19 individuals and healthy controls in indicated immune cell subsets and canonical pathways inferred by Ingenuity pathway analysis (IPA) from DEGs between COVID-19 individuals and healthy controls in indicated immune cell subsets. Red and blue denote different row z-score among nM, nS, and vS.

Supplementary Figure 3
**(A)** Bar diagram indicating the total number of upregulated (white) and downregulated (dark) genes in indicated cell populations from the vS relative to the nS individuals (|FC| = 0.5, nominal p< 0.01). **(B)**Common Canonical pathways inferred by IPA from DEGs between the vS and the nS group in indicated immune cell subsets. Red and blue denote functional pathways predicted to be up or downregulated, respectively by predicted z-score.

Supplementary Figure 4 Transcriptional signatures of immune cell subpopulations during naturally acquired COVID-19 diseases. **(A)** Heatmaps demonstrating transcriptional patterns of Interferon stimulated genes (ISGs) between COVID-19 individuals and healthy controls in indicated immune cell subsets. **(B)** ISGs enrichment analysis was performed using NetworkAnalyst by selecting Reactome pathways; all significant pathways (p<0.05) were listed and grouped according to their functions highlighted in different colors for each cell type.
