# Supplementary material for: Immune-profiling of SARS-CoV-2 viremic patients reveals dysregulated innate immune responses
Source: Front Immunol. 2022 Nov 9;13:984553. doi: 10.3389/fimmu.2022.984553 (PMC9682031; doi:10.3389/fimmu.2022.984553)
Supplement: Supplementary Figure 1 — (A)The flow cytometry gating strategy of seven population and purity. (B) the expression level of putative SARS-CoV-2 entry receptors, including ACE2, CD13, CD26, CD147, CD249, and TMRPSS2, were accessed by flow cytometry. The left panel shows the representative histogram plots of antibody staining gating on monocytes. A Fluorescence Minus One (FMO) control showed in blue and the sample in red. The right panel summarizes each receptor expression level in different cell types (n=20). [file DataSheet_1.pdf]

Figure S1

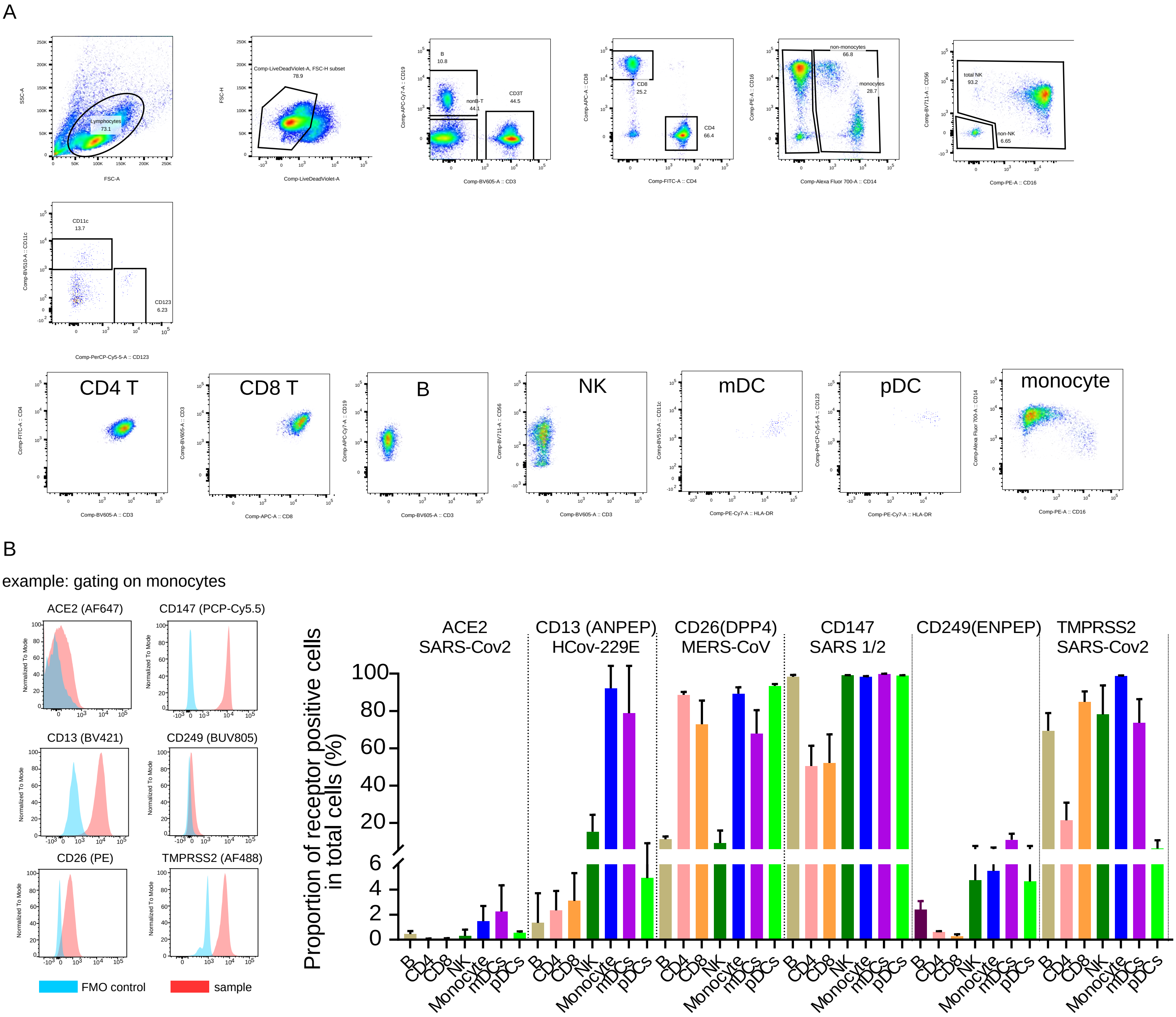

A
Lymphocytes
73.1
Comp-LiveDeadViolet-A, FSC-H subset
78.9
B
10.8
nonB-T
44.1
CD3T
44.5
CD8
25.2
CD4
66.4
non-monocytes
66.8
monocytes
28.7
total NK
93.2
non-NK
6.65
CD11c
13.7
CD123
6.23
CD4 T
CD8 T
B
NK
mDC
pDC
monocyte
B
example: gating on monocytes
ACE2 (AF647)
CD147 (PCP-Cy5.5)
CD13 (BV421)
CD249 (BUV805)
CD26 (PE)
TMPRSS2 (AF488)
FMO control
sample
ACE2
SARS-Cov2
CD13 (ANPEP)
HCov-229E
CD26(DPP4)
MERS-CoV
CD147
SARS 1/2
CD249(ENPEP)
TMPRSS2
SARS-Cov2
Proportion of receptor positive cells in total cells (%)
B
CD4
CD8
NK
Monocyte
mDCs
pDCs

Figure S2

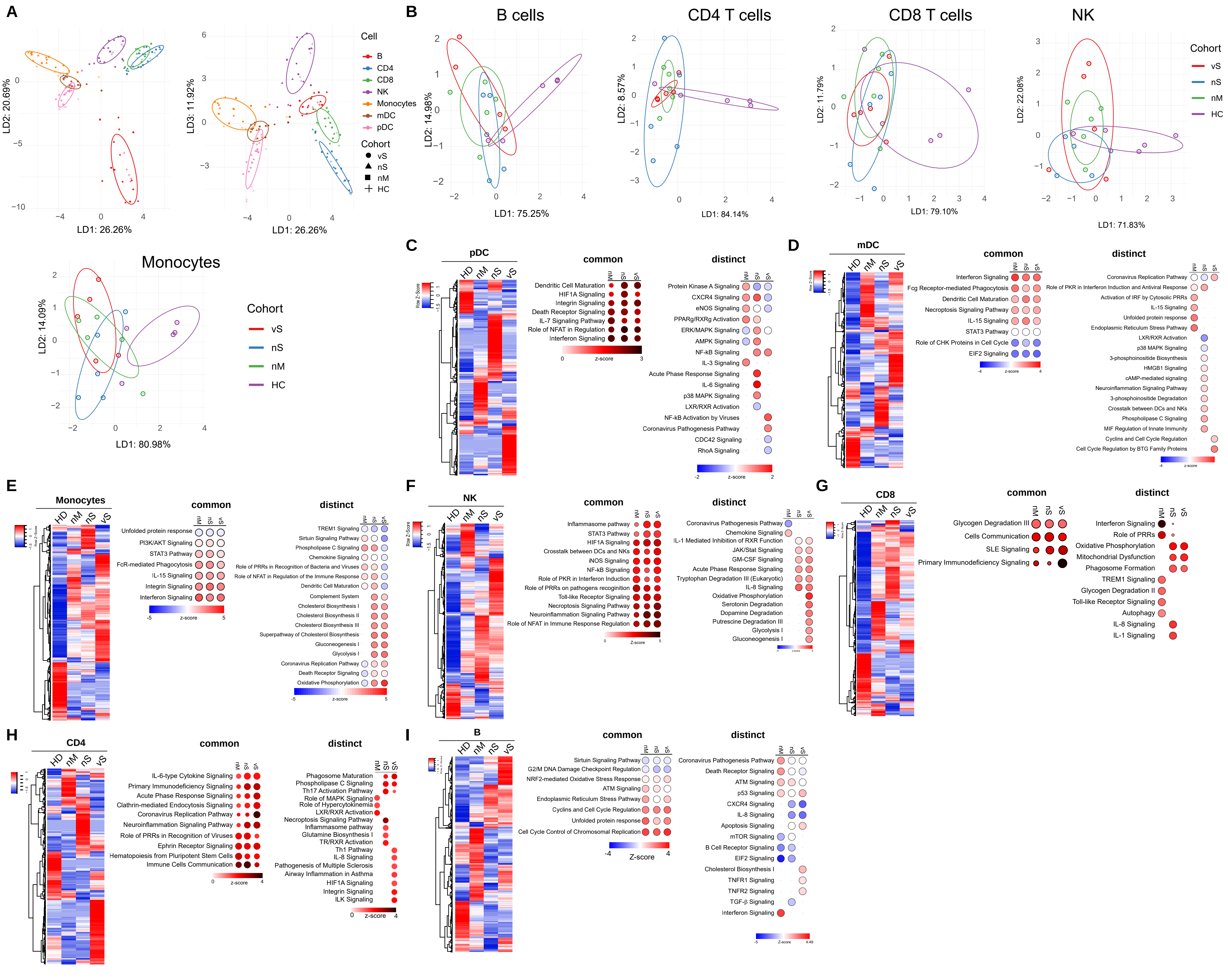

A
B
B cells
CD4 T cells
CD8 T cells
NK
Cell
B
CD4
CD8
NK
Monocytes
mDC
pDC
Cohort
vS
nS
nM
HC
LD1: 26.26%
LD2: 20.69%
LD3: 11.92%
LD1: 75.25%
LD2: 14.98%
LD1: 84.14%
LD2: 8.57%
LD1: 79.10%
LD2: 11.79%
LD1: 71.83%
LD2: 22.08%
Monocytes
LD1: 80.98%
LD2: 14.09%
C
pDC
common
distinct
D
mDC
E
Monocytes
F
NK
G
CD8
H
CD4
I
B
HD
nM
nS
vS
z-score

Figure S3

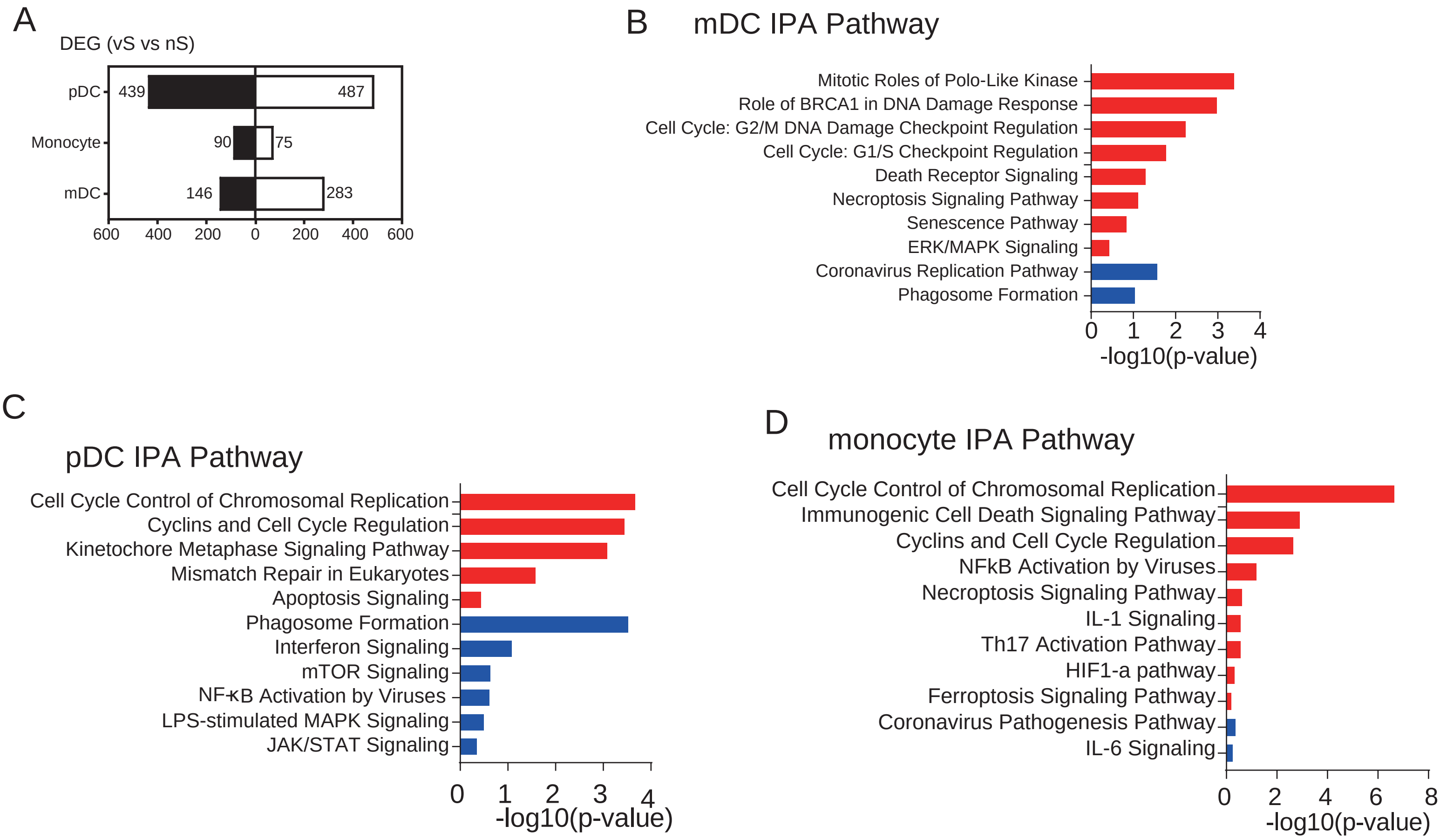

A
DEG (vS vs nS)
pDC
Monocyte
mDC
439
487
90
75
146
283
600
400
200
0
200
400
600
B
mDC IPA Pathway
Mitotic Roles of Polo-Like Kinase
Role of BRCA1 in DNA Damage Response
Cell Cycle: G2/M DNA Damage Checkpoint Regulation
Cell Cycle: G1/S Checkpoint Regulation
Death Receptor Signaling
Necroptosis Signaling Pathway
Senescence Pathway
ERK/MAPK Signaling
Coronavirus Replication Pathway
Phagosome Formation
0 1 2 3 4
-log10(p-value)
C
pDC IPA Pathway
Cell Cycle Control of Chromosomal Replication
Cyclins and Cell Cycle Regulation
Kinetochore Metaphase Signaling Pathway
Mismatch Repair in Eukaryotes
Apoptosis Signaling
Phagosome Formation
Interferon Signaling
mTOR Signaling
NF-κB Activation by Viruses
LPS-stimulated MAPK Signaling
JAK/STAT Signaling
0 1 2 3 4
-log10(p-value)
D
monocyte IPA Pathway
Cell Cycle Control of Chromosomal Replication
Immunogenic Cell Death Signaling Pathway
Cyclins and Cell Cycle Regulation
NFkB Activation by Viruses
Necroptosis Signaling Pathway
IL-1 Signaling
Th17 Activation Pathway
HIF1-a pathway
Ferroptosis Signaling Pathway
Coronavirus Pathogenesis Pathway
IL-6 Signaling
0 2 4 6 8
-log10(p-value)

Figure S4

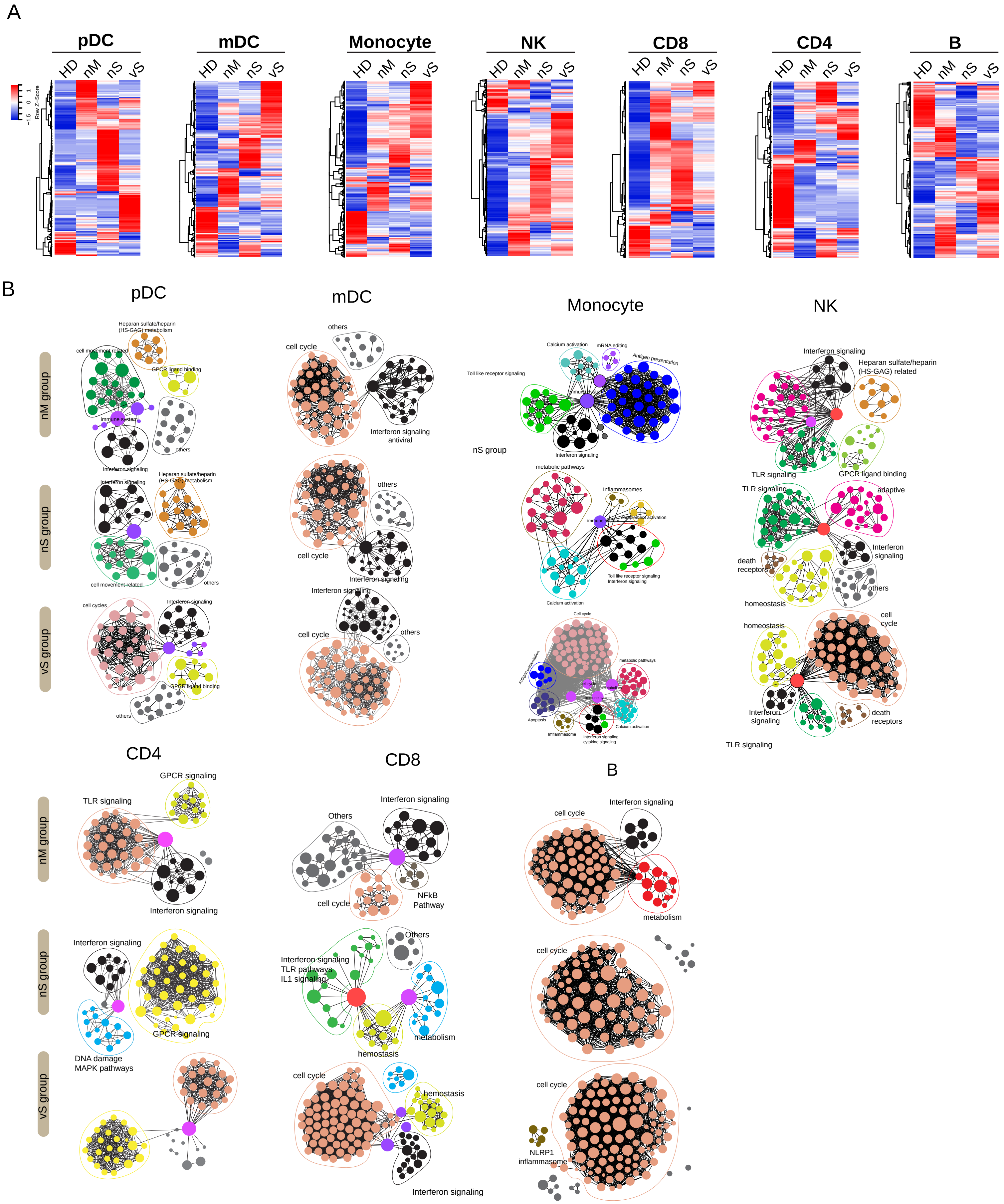
A
pDC
mDC
Monocyte
NK
CD8
CD4
B
HD
nM
nS
vS
Row Z-Score
B
pDC
mDC
Monocyte
NK
nM group
nS group
vS group
cell cycle
others
Interferon signaling
antiviral
Interferon signaling
Heparan sulfate/heparin
(HS-GAG) related
TLR signaling
GPCR ligand binding
adaptive
death
receptors
homeostasis
nS group
CD4
CD8
GPCR signaling
TLR signaling
Others
NFkB
Pathway
metabolism
Interferon signaling
TLR pathways
IL1 signaling
hemostasis
DNA damage
MAPK pathways
NLRP1
inflammasome

Table S1: clinical and demographic information of COVID-19 individuals

	nM	nS	vS	HD
**N**	5	5	5	5
**Age**	59 (42-83)	56 (43-78)	60 (34-84)	56 (35-69)
**Ethnicity**	White 100%	White 100%	White 100%	White 100%
**Gender No. (% female)**	2 40%	2 40%	2 40%	2 40%
**Days Sampling to Symptom onset**	15 (10-19)	14 (11-17)	14 (11-18)	NA
**Fever**	100%	100%	100%	0%
**Cough**	80%	100%	100%	0%
**shortness of breath**	80%	100%	100%	0%
**hypoxia**	60%	100%	100%	0%
**BMI**	29.79 (28.1-36.2)	25.2 (24.3-28.42)	25.7 (21.9-34.1)	26.71 (21.06-34.34)
**Active cancer**	N	N	N	N
**Chronic lung disease**	N	N	N	N
**Hypertension No. (%)**	4 (80%)	3 (60%)	5 (100%)	3 (60%)
**Diabetes No. (%)**	2 (40%)	1 (20%)	4 (80%)	2 (40%)
**Glucose (mg/dL)**	118.6 (97-155)	120 (87-162)	123.4 (90-151)	114.8 (93-153)
**Oxygenation status**	Room air	Nasal cannula	Nasal cannula	NA
**hospitalization status**	Yes	Yes	Yes	NA
**Recovery status**	discharged	discharged	discharged	NA
